# Le syndrome des cheveux étrangleurs: à propos d'un cas

**DOI:** 10.11604/pamj.2019.33.266.15699

**Published:** 2019-07-29

**Authors:** Fatima-Zahra Agharbi

**Affiliations:** 1 Hôpital Civil Tétouan, Tétouan, Maroc

**Keywords:** Cheveux, étrangleurs, tourniquet, Hair, stranglers, tourniquet

## Abstract

The syndrome of the tourniquet or “strangler hair” is the consequence of the strangulation of an appendage or body end by a constrictor link. Toes and fingers are the most affected extremities but also the genitals whether it is the penis, small or large lips or clitoris. Most of the links are hair, more rarely textile fibers (cotton, wool). The speed of diagnosis is important in order to proceed to the removal of the link as soon as possible and avoid seeing the process evolve towards necrosis or inflammation. Spontaneous or surgical amputation of the ischemic extremity. The difficulty lies in the fact that inflammatory edema following progressive constriction is a slow process that can last for weeks. The edema participates in the burying of the link making it accessible to the eye, delaying its ablation by increasing the risk of bone damage, necrosis and partial or total amputation. At the beginning of the process, incessant crying can summarize the symptomatology present by the infant. Different methods have been described to remove the link: mechanical or surgical removal. We report the case of an infant who presented edema and painful erythema of abrupt installation of the third left toe outside a traumatic context. The radiograph did not objectify a fracture or other abnormality and the exploration found constriction by the mother's hair. The evolution was favorable after ablation of the hairs and application of the healing creams.

## Image en médecine

Le syndrome du tourniquet ou du «cheveu étrangleur» est la conséquence de la strangulation d'un appendice ou d'une extrémité corporelle par un lien constricteur. Orteils et doigts sont les extrémités les plus atteintes mais aussi les organes génitaux qu'il s'agisse du pénis, des petites ou grandes lèvres ou du clitoris. La plupart des liens sont des cheveux, plus rarement des fibres textiles (coton, laine). La rapidité de diagnostic est importante afin de procéder à l'ablation du lien dans les plus brefs délais et éviter de voir le processus évoluer vers la nécrose ou l'amputation spontanée ou chirurgicale de l'extrémité ischémiée. La difficulté réside dans le fait que l'œdème inflammatoire consécutif à la constriction progressive est un processus lent qui peut durer des semaines. L'œdème participe à l'enfouissement du lien le rendant accessible à la vue, retardant son ablation en augmentant le risque d'atteinte osseuse, de nécrose et d'amputation partielle ou totale. Au début du processus, des pleurs incessants peuvent résumer la symptomatologie présente par le nourrisson. Différentes méthodes ont été décrites pour retirer le lien: ablation mécanique ou chirurgicale. Nous rapportons l'observation d'un nourrisson qui présentait un œdème et érythème douloureux d'installation brutale du troisième orteil gauche en dehors d'un contexte traumatique. La radiographie n'objectivait pas de fracture ni autre anomalie et l'exploration trouvait une constriction par des poils de la mère. L'évolution était favorable après ablation des poils et application des crèmes cicatrisantes.

**Figure 1:**
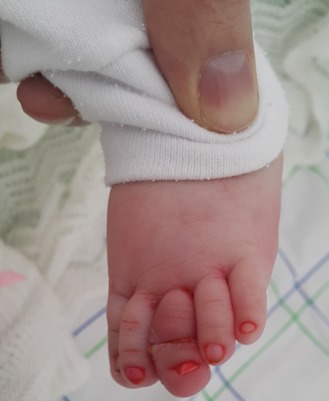
Oedème avec étranglement du troisième orteil gauche

